# Hierarchical Anomaly Detection Model for In-Vehicle Networks Using Machine Learning Algorithms

**DOI:** 10.3390/s20143934

**Published:** 2020-07-15

**Authors:** Seunghyun Park, Jin-Young Choi

**Affiliations:** School of Cybersecurity, Korea University, Seoul 02841, Korea; cloakingmode@korea.edu

**Keywords:** controller area network, intrusion detection system, in-vehicle network security, machine learning, hierarchical approach, anomaly detection, MLHC

## Abstract

The communication and connectivity functions of vehicles increase their vulnerability to hackers. The unintended failure and malfunction of in-vehicle systems caused by external factors threaten the security and safety of passengers. As the controller area network alone cannot protect vehicles from external attacks, techniques to analyze and detect external attacks are required. Therefore, we propose a multi-labeled hierarchical classification (MLHC) intrusion detection model that analyzes and detects external attacks caused by message injection. This model quickly determines the occurrence of attacks and classifies the attack using only existing classified attack data. We evaluated the performance of the model by analyzing its learning space. We further verified the model by comparing its accuracy, F1 score and data learning and evaluation times with the two layers multi-class detection (TLMD) and single-layer multi-class classification (SLMC) models. The simulation results show that the MLHC model has the highest F1 score of 0.9995 and is 87.30% and 99.92% faster than the SLMC and TLMD models in terms of detection time, respectively. Consequently, the proposed model can classify both the type and existence or absence of attacks with high accuracy and can be used in interior communication environments of high-speed vehicles with a high throughput.

## 1. Introduction

High connectivity and automotive electronics are two major developments in modern vehicles, which are evolving to provide various convenience features to drivers. Vehicle connectivity using smart devices and cellular network has enabled the consumption of various contents in the vehicle through an infotainment platform. Particularly, vehicle-to-vehicle communication has enabled the sharing of driving information and dangerous situations on the road. Likewise, vehicle-to-infrastructure communication has broadened the prospects of autonomous vehicles, which have depended on existing sensors only, through the exchange of traffic signals and flows. Furthermore, vehicles are evolving to giant smart devices by being equipped with safety devices, such as forward collision-avoidance and lane-keeping assists, as well as convenience devices, such as telematics and power supply electric devices.

However, such diverse connectivity of vehicles increases their points of attack and exposure to external attacks. As the current controller area network (CAN) message frame lacks authentication or access control mechanisms, in-vehicle data transfer is performed without the use of security techniques. Furthermore, as the in-vehicle controllers are interconnected, the complexity of the architecture increases. The interferences or mutual effects between controllers may cause unintended motions or failures, thus posing further threats to the cybersecurity of vehicles or the safety of passengers.

Existing connected vehicles attain security by configuring a separate dedicated network for in-vehicle Internet services, such as telematics, and separating the connectivity services of the vehicle from the Internet. However, the dedicated network is costly to construct and operate, and it has limitations in opening the platform to expand connectivity-related services. Hence, a more fundamental solution to protect the devices without depending on the traditional communication network security is now required because dedicated Internet services and local area network system have been combined.

To design the cybersecurity of a mission-critical environment, such as vehicles, the characteristics of the external network environment, such as vehicle domain and machine-to-machine (M2M) communication, should be considered. Particularly, intrusion detection or prevention systems of in-vehicle network protection require high accuracy. If important messages in the vehicle are mistaken for an attack and blocked, the vehicle may malfunction and develop safety problems. Therefore, false alarms must be prevented in the intrusion prevention of in-vehicle networks.

Additionally, real-time response is critical for the cybersecurity of vehicles. Malicious attacks on moving vehicles are directly linked to the safety of passengers, pedestrians and other vehicles. Therefore, when external attack messages are identified, the vehicle must be able to implement response measures in real time. However, due to the nature of embedded environments, such as vehicles, there are constraints in temporal and spatial resources. As the available resources for learning and classifying intrusion data are limited, a real-time intrusion detection system (IDS) having high accuracy should be constructed, and it should be able to function with the minimum available computing power of the vehicle.

In 2015, a Jeep Cherokee was remotely hacked and reported to raise awareness of the cybersecurity of vehicles [1]. In a recent article [2], the author suggested that we should not only depend on defending against attacks because it is impossible to produce vehicles with perfect security system to disable hacking, but we should also design the security system to detect attacks and respond appropriately.

Therefore, in this study, we developed a model for detecting anomalous behaviors and attacks caused by message injection on vehicles in real time with high accuracy. We applied a hierarchical data analysis technique for detecting and classifying attack data. Furthermore, to train the intrusion detection model, we minimized misdetections and no-detections using a machine learning algorithm. An appropriate algorithm for the dataset was selected to detect the attack data, and a simulation environment was set up to derive the optimal hyperparameters. Particularly, we propose a method to quickly detect the existence or absence of attacks hierarchically by learning the behaviors of the CAN data. The accuracy of the model was increased to make it applicable to an actual vehicle environment, and a model with real-time responsiveness and using limited resources was implemented. Accuracy, F1 score and detection time were applied as valid metrics to evaluate the proposed model. Using these metrics, we obtained an improved model to detect attacks and anomaly behaviors that flowed into vehicles. The contributions of this study are as follows.

- This is the first study that presents a hierarchical data analysis model for simultaneously classifying the presence or absence of an attack, an attack type and a vehicle type to detect anomaly behaviors in vehicles.
- We present a detection model that includes hyperparameters and an optimal classification algorithm for detection.

The rest of this paper is organized as follows. Section 2 introduces existing related studies. Section 3 details the CAN message frame and topology for an understanding of vehicle cybersecurity. Section 4 describes the dataset we used, as well as the concrete data analysis method and analysis model proposed in this paper. This includes the algorithm for vehicle data analysis, performance measurement metrics and hypothesis space comparison of models for in-vehicle data analysis. Section 5 interprets the simulation results and verifies the effectiveness of the proposed method by comparing it with existing results. In Section 6, we present the conclusion and future research direction.

## 2. Related Work

This section highlights existing works related to this study. The problems in each domain, existing methods to solve them, advantages and disadvantages of the solutions and constraints are stated.

Song et al. [3] proposed an intrusion detection model that learns the sequential pattern of in-vehicle network traffic and detects message insertion attacks according to traffic changes. The structure of the inception-ResNet model designed for large-scale images was used, and the deep convolutional neural network was redesigned by reducing the architecture complexity. Particularly, the authors experimented with a dataset extracted from actual vehicle environment and suggested that detecting complex, irregular random attacks has an advantage. The experiment compared long short-term memory (LSTM), artificial neural network, support vector machine, k-nearest neighbors (kNN) [4], naïve Bayes (NB) and decision tree (DT) [5] algorithms. Zhang et al. [6] proposed a vehicle intrusion detection model based on the neural network algorithm. They compared detection performances using gradient descent with momentum and adaptive gain, and they performed verification and evaluation by applying data collected from actual vehicles. Further, the authors proposed a host-type intrusion detection model for in-vehicle intrusion detection. However, host-type IDS may be inefficient in a broadcast-type communication environment, such as CAN. This architecture is impractical in an embedded environment using limited resources as duplicate detections are performed because every controller receives the same message, and each controller must secure separate resources for intrusion detection. Kang et al. [7] proposed a deep neural network (DNN)-based IDS to monitor the CAN message frame. The DNN model was pre-trained using a deep-belief network. The authors used probability-based feature vectors extracted from packets in learning and training to classify messages as normal or attack. The experiment demonstrated that an accurate detection ratio of approximately 0.98 can be provided in real-time response.

Hoppe et al. [8] placed an anomaly-based IDS in the CAN bus to monitor network traffic. The IDS detects randomly manipulated messages by comparing them with normal patterns. Four attack scenarios related to the CAN bus were presented and classified using the established computer emergency response team taxonomy. It includes technical and managerial considerations to protect the in-vehicle network in comparison with the traditional information technology system, and the countermeasures are discussed by analyzing security vulnerability and potential safety implications. Taylor et al. [9] suggested an anomaly detection method based on the LSTM neural network to detect attacks on the CAN bus. The authors analyzed data by manipulating the identifiers (IDs) of the message frame in a dataset extracted from vehicles rather than infusing attack traffic into the in-vehicle network. By assuming that the CAN traffic was regular, they detected traffic outside the normal sequence in five dataset manipulation scenarios. The result of detecting the known attacks of the CAN bus showed potential for development and provided follow-up tasks to improve the experimental method and detection model. Wang et al. [10] proposed a distributed anomaly detection framework using hierarchical temporal memory (HMM) to strengthen the security of the in-vehicle CAN bus. This method evaluates the output using an abnormal score mechanism that learns the prior state of the CAN network and predicts the flow data. The authors extracted CAN traffic and modified the data fields manually. In addition, they created attack data by replaying the captured traffic on the dataset. They claimed that the area under the curve score was higher than those of the recurrent neural network and HMM, but a method of efficiently detecting attacks where multiple IDs interact without relying on a single message ID should also be considered. Furthermore, experiments are required on indices related to time or resource utilization to examine the applicability of the proposed model to an actual vehicle environment.

The common limitation of the studies mentioned above is that the existing models only determine whether the attack, which is injected in the in-vehicle network, has occurred. In an actual vehicle environment, merely distinguishing between an attack and benign status is insufficient. It is highly important to provide additional information for immediately determining the target affected by the type of attack. It may be easy to inject the attack data in a network and track the sign of occurrence. However, a large amount of computation, which is proportional to the number of target labels, is required to extensively determine the semantics of the attack injected into the vehicle. To address these limitations and satisfy the requirements of an IDS in an actual vehicle environment, we propose a learning model that can not only determine whether an attack occurred, but also classify the attack type and target vehicle.

## 3. In-Vehicle Network Security

To define the proposed multi-labeled hierarchical classification (MLHC) model, this section describes the vehicle CAN message frame, CAN bus structure and attack vector for the vehicle.

### 3.1. Controller Area Network Message Frame and Topology

The CAN is the most representative in-vehicle network technology developed by Robert Bosch GmbH [11] in the early 1980s. Its specifications are still being expanded as a major protocol was used in On-Board Diagnostics II standard. The International Organization for Standardization (ISO) standardized the CAN by ISO 11898 [12] and is still expanding it. This standard was designed to enable communication between in-vehicle microcontrollers and devices and is used for information exchange between electronic control units (ECUs). The CAN device transfers data in packets in message frame units on the CAN network. The message frame does not contain the source or target addresses but only the IDs related to priorities. The real-time priority-based message transfer system follows IDs composed of an 11- or 29-bit string, and a lower ID has a higher priority. First, whether the CAN bus is in use is determined before sending a message to the CAN node, and then collision between messages is detected. When two nodes send a message simultaneously, the message with a higher priority is first sent, and then the message with a lower priority is delayed.

**Figure 1:**
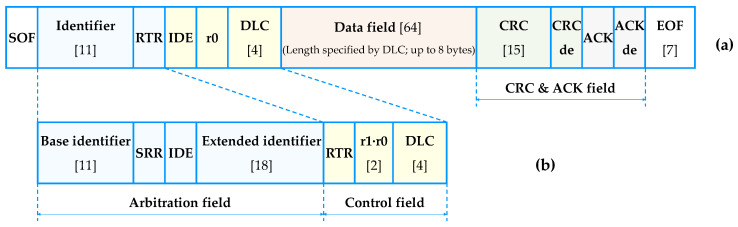
Controller area network (CAN) message structures: (**a**) base format; and (**b**) extended format.

The CAN message frame is divided into base and extended formats depending on the length of the arbitration field, as shown in Figure 1. The base format supports the CAN 2.0A protocol, whereas the extended format supports the CAN 2.0B protocol, and it also accepts the CAN 2.0A protocol. We describe the fields used in the present paper, and the abbreviations for the remaining fields are presented in the Abbreviation Section.

- Base identifier (11 bits): This is the first part of the identifier that indicates the priority of message frames and commonly exists in the standard and extended frames.
- Data length code (DLC, 4 bits): DLC expresses the byte length of the data field in the message frame.
- Data field (64 bits): This is a payload for loading actual data to be sent from one node to the other; a maximum of 8 bytes can be used.

The ECU is a component of the in-vehicle network. It is an embedded device that controls other in-vehicle controllers or devices. The ECU contains input and output interfaces for interconnecting the microcontroller unit, memories (such as read-only and random-access memories), sensors and actuators. The ECU collects and analyzes data from sensors, and it generates control signals and sends them to actuators.

**Figure 2:**
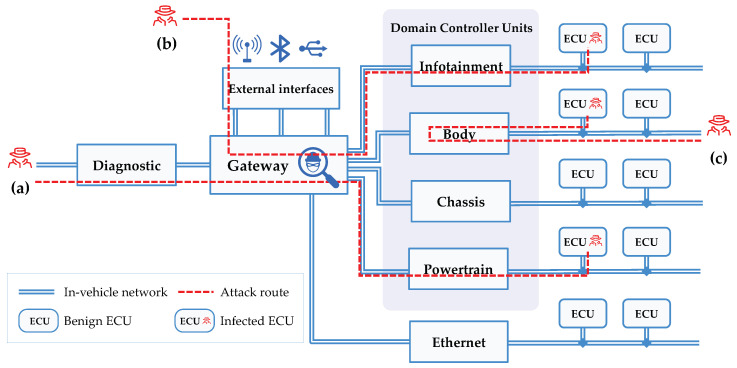
CAN topology and attack vectors: (**a**) external interfaces; (**b**) diagnostic bus; and (**c**) occupation of CAN bus.

Figure 2 illustrates the CAN topology composed of the in-vehicle network and controllers. The ECUs are grouped as the domain controller for logically distinguishing vehicle functions by use, and the CAN bus enables mutual cooperation or control between the ECUs by interconnecting them. Vehicle ethernet may be used for interconnecting controllers that require high-speed communication, and the media-oriented systems transport network is often used for multimedia communication. A gateway may be installed to control diagnostic communication or external interfaces and installing an IDS function for monitoring the CAN traffic inside this gateway may be effective. As shown in Figure 2, external attacks may be injected through a diagnostic bus connected to the CAN bus or an external interface, and this can aid hacking by dominating the CAN bus or ECU.

### 3.2. Attack Vectors on In-Vehicle Network

Attack vectors of confidentiality, integrity and availability aspects need to be considered for defense against vehicle cyberattacks. Attackers can seize the rights for a vehicle or the systems connected to a vehicle and randomly tap major traffic in the vehicle or peek into sensitive information, such as the location of the vehicle. They can also attempt to launch a denial-of-service attack to manipulate the ECU software by reprogramming it. Additionally, they can generate large-scale traffic inside the vehicle to disable normal messages. By entering the in-vehicle network and injecting random messages, hackers can threaten the confidentiality, integrity and availability of the vehicle. Threats of compromising the security objectives of in-vehicle systems are outlined in Table 1.

A monumental event in vehicle cybersecurity occurred in 2015 when Miller and Valasek [1] hacked Jeep Cherokee and opened it to the media and at a hacking conference. They demonstrated a hacking attack targeted at a real moving vehicle by using the vulnerabilities of the cellular network and external interface of the connected service. They accessed the CAN bus through the head unit of a remote vehicle and successfully updated a tampered firmware by acquiring the rights of the controller. After acquiring the control rights of the vehicle, they could remotely operate not only the audio and wiper of the moving vehicle, but also the brakes and steering wheel. Consequently, Fiat Chrysler Automobiles recalled 1.4 million vehicles that could be attacked and was fined $105 million. Furthermore, Tencent’s Keen Security Lab [25] recently seized the rights of a Lexus NX300 using the vulnerability of the audio-video navigation system in the vehicle. They informed the manufacturer that they invaded the CAN bus and successfully injected a malicious message that can cause the vehicle to malfunction and warned of the vulnerability on their blog.

Various attack vectors that may damage the security objectives of vehicles in an in-vehicle network topology are shown in Figure 2. Various remote-connection external interfaces such as Wi-Fi hotspot and Bluetooth are used, as well as the Internet and cellular networks. It is also possible to form sessions with remote vehicles by scanning the M2M network of a specific communication service provider for connectivity services and searching the Internet protocol address and open service ports of the vehicle. In addition, the controller can be operated by force or reprogrammed using diagnostic communication that bypasses the authentication system of the gateway in an in-vehicle network. Once a specific controller is seized, it is possible to launch an attack to occupy the network and stop services by sending many CAN messages with manipulated priorities to the CAN bus.

## 4. Materials and Methods

### 4.1. Multi-Labeled Hierarchical Classification (MLHC) Process

**Figure 3:**
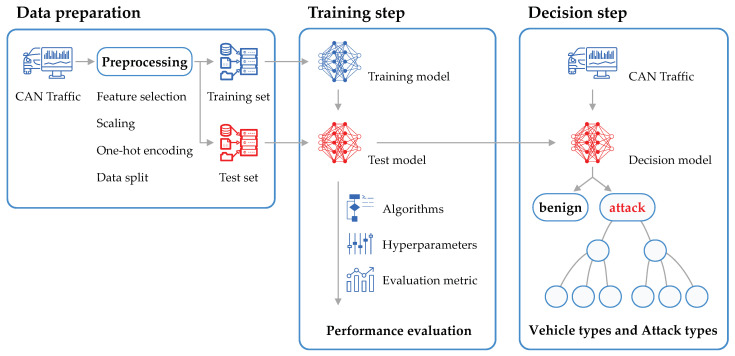
Overall multi-labeled hierarchical classification (MLHC) process.

The overall process of the proposed model is illustrated in Figure 3. The CAN traffic extracted from vehicles is preprocessed to enable the classifier to learn and evaluate it. The data analysis model uses a classification algorithm, preconfigured hyperparameters and performance evaluation metrics. The analysis model is trained by injecting training data, and the performance of the trained model is evaluated using test data. The intrusion detection module, including the trained model in an actual application environment, is used to detect follow-up information, such as attack or benign, vehicle type and attack type, after receiving the CAN message frame as input.

#### 4.1.1. Dataset

The scheme of the in-vehicle network intrusion detection challenge dataset released by Han et al. [27] included CAN ID, DLC and data payload, reflecting the CAN message structure; the timestamp when each data sample was recorded was added into this dataset. They also added a binary label to indicate whether it corresponds to an attack or benign status, whether the data sample is that of an attack or a normal state. We selected this dataset because it includes data extracted from an actual vehicle environment and allows a hierarchical structure of detailed data in the lower layers, such as attack type and vehicle type, for training the vehicle IDS model. The dataset comprises a total of 12 files, with three types of attack data and three vehicle types in normal and message-injected states. This dataset was constructed using data from vehicle models from three vehicle manufacturers. Furthermore, a group of vehicles using the same CAN database formed a vehicle type, and this depended on the vehicle manufacturer that designs the CAN databases. The distributions of the data in each data type are outlined in Table 2.

The message injection into the in-vehicle network was attempted in three attack types as follows. For the flooding attack, several messages were injected with a high-priority CAN ID to induce service delay. For the fuzzing attack, random CAN IDs were injected in brute force until the pre-defined valid CAN ID in the vehicle reacted. For the malfunction attack, valid CAN IDs for each vehicle type were collected in advance, random data fields were configured using the IDs and tampered values were injected. The dataset can be expanded without limitation when additional information is required, such as attack type and vehicle type.

#### 4.1.2. Data Preprocessing

**Figure 4:**
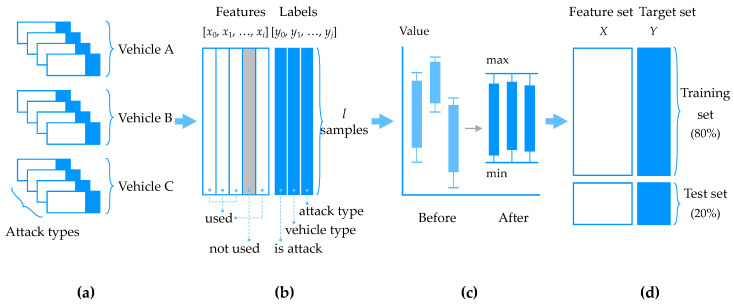
MLHC data preprocessing: (**a**) initial dataset; (**b**) merging and feature selection; (**c**) scaling; and (**d**) data split.

For the classifier to learn the CAN traffic for data analysis, the data preprocessing step illustrated in Figure 4 is required. The CAN IDS dataset used in this model consists of 12 files, which are separated by vehicle type and attack type, and only attack or benign is expressed by binary classification. However, as the vehicle type or attack type is not classified in advance in an actual environment, the intrusion detection module should be able to detect anomalies, even in an environment of random combinations of vehicle types or attack types. Therefore, in this model, to enable the classification of vehicle type and attack type from the incoming data, each unit dataset was integrated into one data frame as shown in Equation (1):

(1) $$S=\sum_{v_{type}}\sum_{a_{type}}S_{v_{type},a_{type}}$$

where *S* is the total dataset required for data analysis, $v_{type}$ is the vehicle type and $a_{type}$ is the attack type. The unit dataset $S_{v_{type},a_{type}}$ is subdivided by attack type and vehicle type, and the existing binary codes are encoded in multiple sub-labels to express additional information, such as vehicle type or attack type.

The features of this dataset include timestamp, time interval, CAN ID, DLC and eight data bytecodes for payload. The feature set of the input data is extracted using the improved feature selection (IFS) method proposed by Park and Choi [28]. This method uses correlations and cross-entropy between the features to combine the high values derived from correlation and information gain. It finds both greedy features as well as the ones with the highest correlation. These two vectors are combined to determine the final features from the dataset that are highly correlated and have a strong impact on the classes. Consequently, timestamp is excluded from the original feature set, and the selected features are as follows: time interval, CAN ID, DLC and data payload. Particularly, the data payload is composed of 64-bit strings at the maximum and can be converted to a byte code string of a length specified by the DLC field. Normalization is applied to prevent underflow or overflow that may occur in the learning process and to evenly distribute the impact on each data string of the payload. The eight independent byte strings having the same values of sections from 0 to 255 are converted to eight floating point variables having a value between 0 and 1 using the min-max normalizer with minimum and maximum values as follows:

(2) $$x_{i}^{\prime }=\frac{x_{i}-\min\left(x_{i}\right)}{\max\left(x_{i}\right)-\min\left(x_{i}\right)}=\frac{x_{i}}{2^{8}-1}$$

where $x_{i}^{\prime }$ is a normalized value and $x_{i}$ is an original vector of feature *i*.

**Figure 5:**
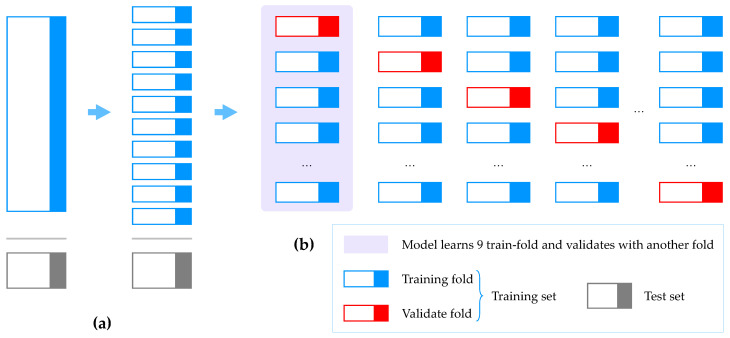
MLHC 10-fold cross validation: (**a**) dividing the training set into 10 folds; and (**b**) learning training-fold and validating with the other fold.

The dataset *S* used as input contains a feature set *X* and target set *Y*. This is split into training, validation and target sets, which are used for learning. For the feature and target sets, *S* is divided into columns, whereas for the training, validation and test sets, S is divided into rows. $x_{i}^{\left(l\right)}$ and $y_{j}^{\left(l\right)}$ denote data elements at feature *i* and labels in the classification group *j* for sample *l*, respectively. In this study, the training and test sets were divided at the ratio 8:2. The model was trained using 80% of the total data, and the performance of the final model was evaluated using the remaining 20% samples. The test set was separated to prevent overfitting and to accurately predict the model performance in a new actual data environment. Notably, the test set was used only for evaluating the model and not for learning. Instead, part of the training data was divided and used for verification to measure the model performance in the learning stage and to obtain hyperparameters yielding excellent performance. This process is illustrated in Figure 5.

After dividing the training set into 10 folds, the model was trained with nine different folds, and the model performance was verified with the remaining fold. The learning was performed 10 times; nine folds were used for training, and the remaining one fold was used for validation.

**Figure 6:**
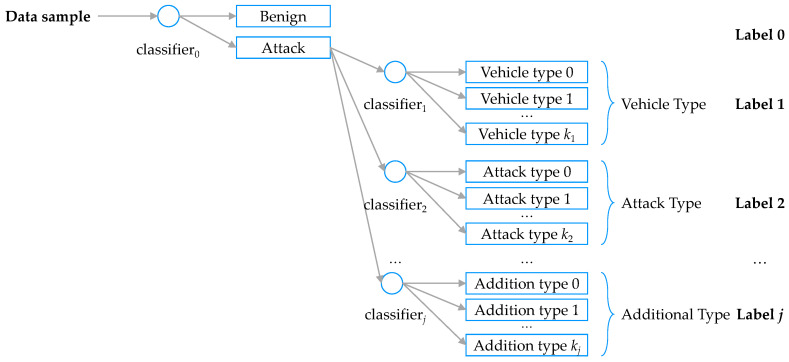
MLHC target labeling.

Additional information must be present in the target data, for example, vehicle information and attack type, as well as the attack or benign of the CAN message. The label was excluded from the feature set for training because it was used to evaluate the learning result in supervised learning. Rather, the label was included in the target data and reorganized to express the additional information, such as vehicle information and attack type, as well as the attack or benign of the CAN message. To hierarchically classify data traffic as suggested in this study, the target data must also form a similar data structure. As shown in Figure 6, the first row of the target data classifies attack or benign, and the lower rows include a hierarchical structure to distinguish the vehicle information or attack type only for attack data. Furthermore, the target data were designed to have a multi-labeled form so that the additional information can be included. Finally, the output data become a vector set including sub-vectors.

### 4.2. MLHC Model

The objective of this study was to effectively detect anomaly behaviors, such as message injection attack, in the CAN traffic of vehicles. To detect intrusion or anomaly behaviors external to the vehicle, an intrusion detection module is required in the CAN bus. Prior studies have detected anomaly behaviors by training normal CAN traffic and analyzing the time interval between messages, or by using machine learning algorithms. In this present study, we adopted a hierarchical approach using multi-label and multi-class classifiers. Hence, we propose a machine-learning-based multi-labeled method for detecting intrusions into the CAN and classifying attack techniques in a hierarchical manner. The multi-class classifier can identify more various categories of data with one classifier as compared to binary classification, and the multi-labeled classifier can contain various types of information simultaneously in a single classifier. This section explains the learning process and algorithm of the hierarchical intrusion detection method using the multi-labeled technique proposed in this study. This subsection describes the MLHC algorithm and compares the space of hypothesis and accuracy according to the classification model.

#### 4.2.1. MLHC Algorithm

The MLHC algorithm and its deployment (see Algorithm 1). The data preprocessing process described in Section 4.1.2 is described on Lines 1–4, and the model learning process is described on Lines 5–17.

In the preprocessing stage, we use the IFS method to select the features for the model (Line 1). Then, we normalize the features using min-max normalization, as described in Equation (2) (Line 2). The training and test sets are split (Line 3); the training set is divided into k folds using k-fold cross-validation (Line 4).

In the learning stage, the algorithm searches through the training data of each training dataset $S_{train}$, determines whether the data sample $x^{\left(l\right)}$ is benign or attack using the first classifier $c_{0}$ and records the result in ${\hat{y}}_{0}^{\left(l\right)}$ (Line 7). If the data sample indicates a benign state, it is not classified further, and the learning of the corresponding sample is terminated (Lines 8–9). Otherwise, ${\hat{y}}_{j}^{\left(l\right)}$ (Line 10), the result of additional classification using the sub-classifier $c_{j}$ is obtained and stored in the detailed information vector ${\hat{V}}^{\left(l\right)}$ (Lines 12–13). $\hat{Y}$, which is returned as the result of the model, is composed of a set comprising ${\hat{y}}^{\left(l\right)}$ as its elements, as shown in Equation (3):

(3) $${\hat{Y}}^{\left(l\right)}=\left\{{\hat{y}}^{\left(l\right)}|l\in \left\{0,1,\ldots ,n(S)\right\}\right\}$$

where *l* is an index of a sample of dataset *S*. Regarding dataset *S*, $S_{train}$ is the training set and $S_{test}$ is the test set. This is generally expressed as *S*. The result for each sample *l* can be expressed as a concatenation of ${\hat{y}}_{0}^{\left(l\right)}$ and ${\hat{V}}^{\left(l\right)}$ (Line 16), as expressed in Equation (4):

(4) $${\hat{y}}^{\left(l\right)}=\left[\begin{array}{cc} {\hat{y}}_{0}^{\left(l\right)} & {\hat{V}}^{\left(l\right)} \end{array}\right]$$

where ${\hat{y}}_{0}^{\left(l\right)}$ is a binary classification result to determine whether sample *l* is a benign or an attack case. ${\hat{V}}^{\left(l\right)}$ is a vector set that expresses additional information if ${\hat{y}}^{\left(l\right)}$ is an attack, and it can be expressed in detail as Equation (5):

(5) $${\hat{V}}^{\left(l\right)}=\left\{\begin{array}{cc} \emptyset , & \mathrm{for}\;{\hat{y}}_{0}^{\left(l\right)}\;\mathrm{is}\;benign\\ \left[\begin{array}{cccc} {\hat{y}}_{1}^{\left(l\right)} & {\hat{y}}_{2}^{\left(l\right)} & \cdots & {\hat{y}}_{j}^{\left(l\right)} \end{array}\right], & \mathrm{otherwise} \end{array}\right.,$$

where ${\hat{V}}^{\left(l\right)}$ is an empty matrix if ${\hat{y}}_{0}^{\left(l\right)}$ is a benign case. On the other hand, ${\hat{V}}^{\left(l\right)}$ has a matrix of elements ${\hat{y}}_{1}^{\left(l\right)}$, ${\hat{y}}_{2}^{\left(l\right)}$, *…*, ${\hat{y}}_{j}^{\left(l\right)}$ representing additional learning results by each classifier if ${\hat{y}}_{0}^{\left(l\right)}$ is an attack.
**Algorithm 1** Multi-labeled hierarchical anomaly detection.**Input:** *S* is a universal dataset including a feature set *X* and a target set *Y*.**Output:** $\hat{Y}$ is a set of learning results including ${\hat{y}}_{0}^{\left(l\right)}$ and ${\hat{V}}^{\left(l\right)}$ for all samples *l*.${\hat{y}}_{0}^{\left(l\right)}$ is a result of determining whether sample *l* is a benign or an attack.${\hat{V}}^{\left(l\right)}$ is a combination of additional classification results for the attack sample (j≠0).1:Select features $x_{i}\in X$ using *Improved Feature Selection*.2:Normalize $x_{i}$ for all features using *min_max normalizer*.3:Split the dataset *S* into a training set $S_{train}$ and a test set $S_{test}$ for $S_{train}\cap S_{test}=\emptyset$.4:Split $S_{train}$ into *k* folds and designate a fold $S_{validation}$ as the validation set excluded from the training set. $n\left(S\right)=n\left(S_{train}\right)+n\left(S_{validation}\right)+n\left(S_{test}\right)$5:**for**$x^{\left(l\right)}\in X\wedge X\subset S_{train}$**do**6: Initialize ${\hat{V}}^{\left(l\right)}\leftarrow \emptyset$7: ${\hat{y}}_{0}^{\left(l\right)}\leftarrow c_{0}\left(x^{\left(l\right)}\right)$
8: **if** ${\hat{y}}_{0}^{\left(l\right)}=benign$ **then**
9:  *// do nothing*
10: **else**
11:  **for** $c_{j}\in C\wedge j\neq 0$ **do**
12:   ${\hat{y}}_{0}^{\left(l\right)}\leftarrow c_{j}\left(x^{\left(l\right)}\right)$
13:   ${\hat{V}}^{\left(l\right)}\leftarrow \mathrm{add}\left(y_{j}^{\left(l\right)}\right)$
14:  **end for**
15: **end if**
16: ${\hat{y}}^{\left(l\right)}\leftarrow {\hat{y}}_{0}^{\left(l\right)}\mathrm{concat}{\hat{V}}^{\left(l\right)}$
17:**end for**

#### 4.2.2. Confusion Matrix and Evaluation Metric for MLHC

A confusion matrix is used to evaluate the classification results. In general, when the training results of the model are returned only in binary classification, the results are expressed in only two types, positive and negative, so they have a simple matrix, as presented in Table 3.

However, the proposed MLHC method contains more information than the typical confusion matrix because it is a multi-class method that processes data of various categories and contains various classification results simultaneously. Similar to the existing confusion matrix, the confusion matrix indicates true negative (TN) or true positive (TP) if the benign sample is classified accurately as benign, or the sub-classification information of the attack sample, such as vehicle type and attack type, is accurately detected. Furthermore, the matrix classifies it as false negative (FN) if attack detection is missed because the sample containing sub attack information is misclassified as normal and as false positive (FP) if normal data are erroneously detected as attack; a sub attack classification result is then returned. The difference from the existing confusion matrix is that if the model classifies a data sample as attack, classification results of various categories are included in the layers below the attack. If the first classifier accurately detected an attack but erroneously classified additional information, such as vehicle type and attack type in the lower layers, it is classified as partial true positive (PTP). The hierarchical confusion matrix that contains PTP in the MLHC model is shown in Table 4.

For the model’s performance, among the accuracy classification indies, accuracy and F1 score are used as shown in Equations (6) and (9), respectively.

(6) $$Accuracy=\frac{TN+\sum TP}{TN+\sum TP+\sum FN+\sum FP+\sum PTP}$$

where accuracy represents the ratio of accurate classification of attack cases as attack and benign cases as benign among all cases. For attack cases, only TP cases where even the additional information type is correct are counted as follows. The precision, which represents the probability that the actual correct answer is included among the values predicted as attack (i.e., $P_{predict}$) by the classifier, is expressed as follows:

(7) $$Precision=\frac{\mathrm{positive}\mathrm{detections}}{\mathrm{whole}\mathrm{detections}\mathrm{of}\mathrm{an}\mathrm{algorithm}}=\frac{\sum TP}{P_{Predict}}=\frac{\sum TP}{\sum TP+\sum FP+\sum PTP}$$

However, precision does not include the PTP cases where the vehicle type or attack type is not accurately detected.

The recall, which represents the probability that the actual attack cases noted as *P* are accurately predicted as attack by the classifier, is expressed as follows:

(8) $$Recall=\frac{\mathrm{positive}\mathrm{detections}}{\mathrm{total}\mathrm{number}\mathrm{of}\mathrm{existing}\mathrm{positives}}=\frac{\sum TP}{P}=\frac{\sum TP}{\sum TP+\sum FN+\sum PTP}$$

As with the precision, PTP cases are not included in recall. Precision and recall have a trade-off relationship with each other. When the recall is raised by adjusting the parameters of the algorithm, false alarms increase; if the conditions are strengthened to reduce false alarms, the recall drops. Therefore, recall and precision should be considered together. Hence, in this study, we used F1 score, which is the harmonic mean of these two items, as follows:

(9) $$F1score=2\times \frac{Precision\times Recall}{Precision+Recall}$$

#### 4.2.3. Space of Hypothesis

The space of hypothesis H(S,C), which represents the space set of the model, product of the number of samples and number of classifiers, increases in proportion to the quotient of the data depth. It can be expressed as Equation (10):

(10) $$H\left(S,C\right)={\left(n(S)\times n(C)\right)}^{depth}$$

where *S* is the set of all samples, *C* is the set of classifiers for distinguishing the type of each target and depth is the number of layers of each classifier. The related notations are outlined in Table 5.

In this section, the existing two models, two-layer multi-class detection (TLMD) and single-layer based multi-class classification (SLMC), are compared in terms of space set with our proposed data learning model MLHC. The TLMD model proposed by Yuan et al. [29] performs multi-class classification independently in each layer by two independent classifiers using the C5.0 algorithm and NB algorithm, respectively. By contrast, the method proposed by Aburomman and Reaz [30] is an SLMC model that contains a multi-class classifier using a support vector machine that has a weight in one layer.

**Figure 7:**
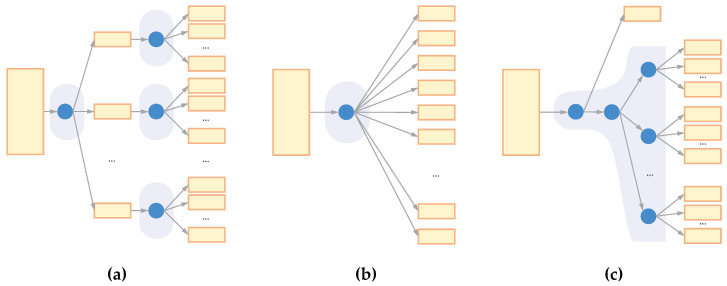
Comparison classification methods: (**a**) two layers multi-class detection model; (**b**) single-layer based multi-class classification model; and (**c**) multi-labeled hierarchical classification model (our approach).

Figure 7a illustrates the traditional model TLMD, which repeats the learning of the total dataset for the number of classifiers, and the computation of TLMD is shown in Equation (11):

(11) $$H_{TLMD}\left(S,C\right)=\frac{n(S)}{c_{0}}\times \frac{n(S)}{c_{1}}\times \cdots \times \frac{n(S)}{c_{j}}={\left(n(S)\right)}^{j+1}\cdot \prod_{i=0}^{j}\left(\frac{1}{c_{i}}\right)$$

where the number of sample data to be learned in each classifier is $n\left(S\right)/c_{j}$, and training is repeated for the number of classifiers $c_{j}$.

Figure 7b illustrates the SLMC for classifying all the target data using one classifier. The multi-class classification method is used because the number of classes $k_{j}$ classified by every classifier *C* must be expressed. The computation of SLMC is expressed as Equation (12):

(12) $$H_{SLMC}\left(S,C\right)={\left\{n\left(S\right)\times \left(c_{0}\times c_{1}\times \cdots \times c_{j}\right)\right\}}^{1}=n\left(S\right)\cdot \prod_{i=0}^{j}c_{i}$$

where the target data are expressed as a combination of all data types that can be expressed by each classifier. Therefore, classifier *C* is $c_{0}\times c_{1}\times \cdots \times c_{j}$, and the depth is one.

By contrast, our proposed MLHC method in Figure 7c forms one classifier by combining multi-class classification and multi-labeled classification. Therefore, the computation of the MLHC is expressed as Equation (13):

(13) $$H_{MLHC}\left(S,C\right)={\left\{n\left(S\right)\times \left(1+c_{1}\times \cdots \times c_{j}\right)\right\}}^{1}=n\left(S\right)\cdot \left(1+\prod_{i=1}^{j}c_{i}\right)$$

Compared to Equation (12), Equation (13) can reduce the amount of computation for benign data because it does not perform a separate classification process if the result of classifier $c_{0}$ of the first layer is benign. To compare them with each other, the two equations are rearranged after replacing $n\left(S\right)\cdot \prod_{i=1}^{j}c_{i}$ with δ. $H_{SLMC}\left(S,C\right)$ and $H_{MLHC}\left(S,C\right)$ are expressed as Equations (14) and (15), respectively:

(14) $$H_{SLMC}\left(S,C\right)=n\left(S\right)\cdot c_{0}\cdot \prod_{i=1}^{j}c_{i}=\delta \cdot c_{0}$$

(15) $$H_{MLHC}\left(S,C\right)=n\left(S\right)+n\left(S\right)\cdot \prod_{i=1}^{j}c_{i}=\delta +n\left(S\right)$$

In the SLMC model, an increase in data types to be classified means that the space of hypothesis increases according to the multiplicative function. By contrast, in the MLHC model, classifier $c_{0}$ of the first layer determines benign or attack; if it is benign, classification stops. Therefore, the amount of computation can be reduced for the amount of benign data. When the present dataset, where 89.39% of the total data is benign, is applied, only 10.61% of the attack data is used to classify the vehicle type and attack type. Hence, the space of hypothesis is reduced for the ratio of attack data.

## 5. Results and Discussion

### 5.1. Simulation Environments

In the simulation, the data were learned using the learning model described in Section 4.2, and the performance was compared by measuring accuracy and time. For the intrusion detection model of the in-vehicle network, we used the dataset [27] released from the challenge of in-vehicle intrusion detection. The model was trained and verified by randomly extracting 80% of the data samples from a total of 1.73 million data samples, and the model performance was evaluated using the remaining 20% of the data samples. To classify attack or benign, vehicle type and attack type of CAN traffic, the data samples were learned as multi-labels, and the targets were classified as multi-classes to accommodate various vehicle types and attack techniques.

We used four machine learning algorithms to compare the performance of the proposed method. The stochastic gradient descent (SGD) algorithm [31] is an iterative algorithm used for optimizing objective functions such that they have suitable smoothness properties. We used SGD in our study to compare the performance of the machine learning algorithms, as it reduces the computational burden associated with high-dimensional optimization problems, thereby achieving faster iterations, although the convergence rate obtained is low. In the kNN classification algorithm, the input consists of the k-closest training examples in the feature space. An object is classified by a plurality vote of its neighbors, with the object being assigned to the class most common among its k nearest neighbors. We used this algorithm in our study, as it is basic and capable of performing multi-class classification for performance evaluation.

The DT algorithm constructs a tree structure where each non-leaf node represents an attribute evaluation and each leaf node represents a class label. This algorithm can effectively analyze and classify the data to identify the attributes with information gain. We also used DT in our study as it is a classification algorithm and can achieve good performance depending on the type of dataset used. Furthermore, the random forest (RF) algorithm [32] is a kind of ensemble learning that is used for classification and regression. It returns the classification and average prediction results from the DTs and is therefore an extension of DT. We used the RF algorithm as well, to address the problem of overfitting on the training data and for obtaining a high accuracy.

To evaluate the performance of the classification model, detection rate and training time were selected as evaluation metrics. Accuracy, recall, precision and F1 score were calculated to evaluate the accuracy of the model in a reliable manner, and the elapsed time for training and evaluation of the model were measured. For the reference to evaluate whether the data samples were accurately classified, we used the hierarchical confusion matrix illustrated in Table 4. This matrix does not include PTPs in TPs where the vehicle type or attack type is incorrect even if the attack or benign is accurately detected. We implemented classifiers using our novel method specified in Algorithm 1 and measured the accuracy.

### 5.2. Simulation Results

**Figure 8:**
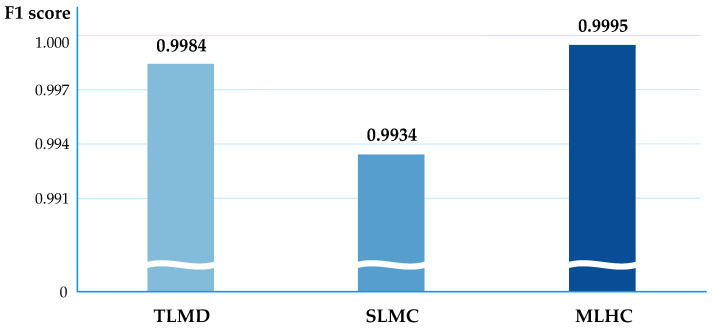
Simulation results for the best F1 score of each model.

Table 6 compares and outlines the simulation results based on the four machine learning algorithms, namely, SGD, kNN, DT and RF, in terms of the detection rate; these models are described in Section 4.2.3. The results are rounded from the fifth decimal place. Among the three models described, the RF algorithm shows a high positive detection rate of 0.99 or higher. Particularly, the MLHC model proposed in this study showed the highest detection rates evenly in the other three algorithms. The algorithm having the highest F1 score in each model and a graph of F1 score are shown in Figure 8. All three models showed the highest performance with RF. If the training time is not considered, it can be seen that the F1 score of the model is the highest in MLHC, followed by TLMD and SLMC. The reason for the higher detection rate of MLHC as compared to the other models can be explained as follows.

MLHC determines whether an attack has occurred and then classifies the attack information in a hierarchical manner. Therefore, benign and attack data are separated for each data sample in the first stage itself. Subsequently, the model uses only the attack data when classifying specific attack information such as the attack type and vehicle type. Therefore, in this model, the benign data do not contribute to any errors. Consequently, it can be seen that the MLHC model shows a higher detection rate than the TLMD model, which contains two layers and the SLMC model, which comprises a single layer.

Table 7 illustrates the measurement result of the time elapsed for training and model evaluation in each model. For the training data, 1,388,672 data samples corresponding to 80% of all data samples were extracted randomly. Each model was evaluated using the remaining 20% (347,168) of the data samples. The first method TLMD uses independent classifiers in each layer to classify the attack type and vehicle type from the CAN traffic data. For this, $\prod_{i}^{j=9}\left(n\left(S\right)/c_{i}\right)$ needs to be computed in the three layers using Equation (11) during training. Consequently, TLMD took the largest amount of time for training and evaluating all the algorithms. In addition, in the case of the SLMC model, the hypothesis space is proportional to the product of the number of sample and the number of classifiers. The hypothesis space is represented by $n\left(S\right)\cdot \prod_{i}^{j=24}c_{i}$ as shown in Equation (12), and the number of classifiers is 24.

On the contrary, the MLHC model uses a classifier to learn the entire data and then determines if a data sample represents a an attack or benign state. In this method, the benign data that do not require additional analysis, such as vehicle type or attack type, are excluded from the sub-classification targets. Therefore, Equation (13) is used to reduce the amount of calculation as many as the number of benign data compared SLMC of Equation (12). Therefore, since in an MLHC model using a single classifier, the benign data (89.4% of the total data) need not be reclassified, 99.92% of the learning time is reduced on average, as compared to the TLMD model.

**Figure 9:**
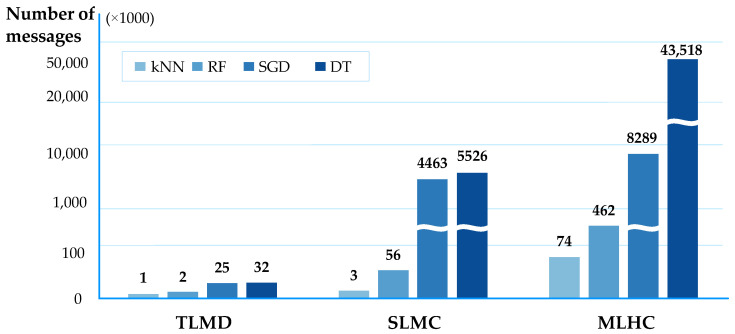
Simulation results: Number of CAN messages that can be processed per second.

Figure 9 shows the number of CAN messages that can be processed per unit time for each algorithm of each model. The kNN and RF of the TLMD model processed 528 and 1927 test messages per second, respectively, whereas the kNN of the SLMC model processed 2973 messages per second. Considering that 1 Mbps of CAN has 50% of channel utilization, 5000 or more messages must be processed per second. Therefore, the three types of models are not suitable for processing the flooding messages in real time. If high-speed CAN communication in the future is considered, the DT algorithm of the MLHC model that can process 43.5 million messages per second should be used to prevent the bottleneck of the intrusion detection module.

## 6. Conclusions

This paper proposes the MLHC learning model that hierarchically classifies attacks using a machine learning algorithm to detect anomaly behaviors of the in-vehicle network accurately and rapidly. The MLHC method can make quick judgements about attack or benign cases for in-vehicle networks by learning the CAN traffic, and it can classify additional detailed information when an attack is detected. A learning model that accommodates multi-labeled multi-class schemas was designed to include various attributes simultaneously while classifying various types of attack data. To evaluate the performance of our model, we applied four machine learning algorithms to existing models and compared accuracy, precision, recall, F1 score and elapsed times for training step and test step.

The simulation results show that the proposed MLHC model achieved high accuracy when based on the RF algorithm and rapid detection when based on the DT algorithm. Both algorithms derived F1 scores higher than 0.998. Thus, we conclude that the DT and RF algorithms are applicable to high-speed internal communication environments, as well as in CAN for analyzing 43 million and 46 million CAN message frames per second, respectively.

In the future, we plan to train and verify intrusion detection models based on traffic injected into vehicles after directly generating messages of various attack types in addition to fuzzing, flooding and malfunction. Furthermore, we will additionally analyze the vehicle ethernet traffic beyond the CAN for target networks to investigate methods of applying the traditional intrusion detection and prevention patterns to the in-vehicle network. In addition, in the future, we intend to investigate the parallel processing method [33] for fast data processing in real time against sequential message injection attacks.

## Figures and Tables

**Table 1:** Summary of security objectives and corresponding threats on in-vehicle network.

Security Objectives	Threats	Related Work	Attack Vectors
**Availability**	Damage of the internal systems by denial-of-service attack (flooding)	[13]	CAN bus, gateway, external interface
	Interference with short-range communication or sensor recognition	[14,15,16]	External interface, sensor
	Unintended service interruption (fuzzing)	[17]	CAN bus, ECU
	Blockage of normal message flow	[13]	CAN bus, gateway
**Confidentiality**	Illegal upgrade or acquisition of rights	[18,19]	ECU, memory
	Access to unauthorized information	[20]	External interface, ECU, memory
	Information leakage by damaged applications (malfunction)	[21]	ECU
	Acquisition of the encryption key by sniffing	[22]	External interface
**Integrity**	Forging and falsification of control messages	[23]	CAN bus, ECU
	Injection of malicious messages and forced operation of the controller (fuzzing)	[24]	CAN bus, gateway, ECU
	Manipulation of the firmware and update with a tampered firmware	[1,25]	ECU
	Installation of backdoor	[26]	ECU

**Table 2:** Statistics of CAN intrusion dataset.

Classification			Total	Vehicle Types		
				Vehicle_A	Vehicle_B	Vehicle_C
**Total**			1,735,840	402,956	535,041	797,843
**Attack types**	**Benign**		1,552,526	366,510	468,527	717,489
	**Attack**	Flooding	88,150	22,587	32,422	33,141
		Fuzzing	63,742	5812	18,118	39,812
		Malfunction	31,422	8047	15,974	7401

**Table 3:** Confusion matrix for binary classification.

		Prediction	
		Positive	Negative
**Actual**	Positive	True Positive	False Negative
	Negative	False Positive	True Negative

**Table 4:** Hierarchical confusion matrix.

				Prediction							
				Attack							Benign
				VT_0			VT_1			⋯	
				AT_0	AT_1	AT_2	AT_0	AT_1	AT_2	⋯	
**Actual**	**Attack**	VT_0	AT_0	TP	PTP	PTP	PTP	PTP	PTP	⋯	FN
			AT_1	PTP	TP	PTP	PTP	PTP	PTP	⋯	FN
			AT_2	PTP	PTP	TP	PTP	PTP	PTP	⋯	FN
		VT_1	AT_0	PTP	PTP	PTP	TP	PTP	PTP	⋯	FN
			AT_1	PTP	PTP	PTP	PTP	TP	PTP	⋯	FN
			AT_2	PTP	PTP	PTP	PTP	PTP	TP	⋯	FN
		⋯	⋯	⋯	⋯	⋯	⋯	⋯	⋯	⋯	⋯
	**Benign**			FP	FP	FP	FP	FP	FP	⋯	TN

**Table 5:** Summary of notations.

Notation	Description
*S*	Set of full datasets which containing benign, attack and attack types extracted from several vehicle models.
$S_{\alpha }$, $S_{\beta }$	Subsets of *S*, each composed of attack and benign samples, respectively. $S=S_{\alpha }\cup S_{\beta }$, $S_{\alpha }\cap S_{\beta }=\emptyset$, $S_{\beta }=\left\{\left(X,Y\right)|y_{0}=0\right\}$.
*l*	Index of sample at the line of *l* in *S*. (0≤l≤n(S)).
$x_{i}$	Features are elements of feature set *X*, and *i* is an index of the feature. $\left(x_{i}\in X\;\mathrm{and}\;i=0,1,\ldots ,I\right)$
$y_{j}$	Types of target are elements of target set *Y*, and *j* is the number of classifiers. Especially if the index *j* of *y* is zero, (i.e., $y_{0}$) is an indicator of whether the target is benign or attack. $\left(y_{j}\in Y\;\mathrm{and}\;j=0,1,\ldots ,J\right)$
${\hat{y}}_{j}$	Predicted target classes are elements of prediction result set $\hat{Y}$. $\left({\hat{y}}_{j}\in \hat{Y}\right)$
$\hat{V}$	Subset of $\hat{Y}$ excluding $y_{0}$ represents multiple predicted types from classifiers. $\left({\hat{y}}_{0}\notin \hat{V},\hat{V}\subset \hat{Y}\;\mathrm{and}\;{\hat{y}}_{0}=0\right)$
$c_{j}$	Classifiers are elements of classifier set *C*, and *j* is an index of classifier. $\left(c_{j}\in C\right)$
$k_{j}$	The number of types on each classifier $c_{j}$.
H(S,C)	Space of hypothesis.

**Table 6:** Simulation results for detection rate.

Evaluation Metric	Algorithm	TLMD	SLMC	MLHC
**Accuracy**	SGD	0.9111	0.9055	0.9336
	kNN	0.9431	0.9924	0.9950
	DT	0.9617	0.9984	0.9997
	RF	0.9989	0.9992	0.9999
**Precision**	SGD	0.9203	0.2877	0.9001
	kNN	0.9228	0.9399	0.9526
	DT	0.9398	0.9915	0.9974
	RF	0.9983	0.9934	0.9993
**Recall**	SGD	0.9065	0.6193	0.9171
	kNN	0.9216	0.9312	0.9573
	DT	0.9416	0.9872	0.9986
	RF	0.9986	0.9934	0.9998
**F1 score**	SGD	0.9133	0.3929	0.9085
	kNN	0.9222	0.9355	0.9550
	DT	0.9407	0.9893	0.9980
	RF	0.9984	0.9934	0.9995

**Table 7:** Simulation results for elapsed time.

Evauation Metric	Algorithm	TLMD	SLMC	MLHC
**Training Time (s)**	SGD	320.871	17.793	2.110
	kNN	4495.222	715.893	4.609
	DT	272.642	11.138	0.618
	RF	1445.077	236.428	13.033
**Test time (s)**	SGD	14.048	0.078	0.042
	kNN	659.283	117.097	4.707
	DT	11.208	0.063	0.008
	RF	180.634	6.170	0.753

## Abbreviations

The following abbreviations are used in this manuscript:

MLHC	Multi-labeled hierarchical classification
TLMD	Two layers multi-class detection
SLMC	Single-layer multi-class classification
CAN	Controller area network
M2M	Machine-to-machine
IDS	Intrusion detection system
LSTM	long short-term memory
kNN	k-nearest neighbors
NB	Naïve Bayes
DT	Decision tree
DNN	Deep neural network
ID	Identifier
HTM	Hierarchical temporal memory
ISO	International Organization for Standardization
ECU	Electronic control unit
SOF	Start of frame
RTR	Remote transmission request
SRR	Substitute remote request
IDE	Identifier extension
DLC	Data length code
CRC	Cyclic redundancy check
ACK	Acknowledgement
EOF	End of frame
IFS	Improved feature selection
TN	True negative
TP	True positive
FN	False negative
FP	False positive
PTP	Partial true positive
SGD	Stochastic gradient descent
RF	Random forest
